# The Role of NK Cells in Pig-to-Human Xenotransplantation

**DOI:** 10.1155/2017/4627384

**Published:** 2017-12-19

**Authors:** Gisella Puga Yung, Mårten K. J. Schneider, Jörg D. Seebach

**Affiliations:** ^1^Laboratory for Translational Immunology, Division of Immunology and Allergy, University Hospital and Medical Faculty, Geneva, Switzerland; ^2^Laboratory for Transplantation Immunology, Department of Internal Medicine, University Hospital Zurich, Zurich, Switzerland

## Abstract

Recruitment of human NK cells to porcine tissues has been demonstrated in pig organs perfused ex vivo with human blood in the early 1990s. Subsequently, the molecular mechanisms leading to adhesion and cytotoxicity in human NK cell-porcine endothelial cell (pEC) interactions have been elucidated *in vitro* to identify targets for therapeutic interventions. Specific molecular strategies to overcome human anti-pig NK cell responses include (1) blocking of the molecular events leading to recruitment (chemotaxis, adhesion, and transmigration), (2) expression of human MHC class I molecules on pECs that inhibit NK cells, and (3) elimination or blocking of pig ligands for activating human NK receptors. The potential of cell-based strategies including tolerogenic dendritic cells (DC) and regulatory T cells (Treg) and the latest progress using transgenic pigs genetically modified to reduce xenogeneic NK cell responses are discussed. Finally, we present the status of phenotypic and functional characterization of nonhuman primate (NHP) NK cells, essential for studying their role in xenograft rejection using preclinical pig-to-NHP models, and summarize key advances and important perspectives for future research.

## 1. Introduction

The field of xenotransplantation explores the feasibility of replacing nonfunctional organs of one species by organs of another species and to overcome the current worldwide organ shortage in transplantation medicine [1]. Within the range of conceivable animals, pigs are the most suitable for xenotransplantation purposes for several reasons [2, 3]. However, before xenotransplantation becomes a clinical reality, many aspects of interspecies immunological and biological incompatibilities need to be taken into consideration [4, 5]. Recent reviews recapitulate the current advances in the field including a summary of the main mechanisms involved in xenorejection and how to control them and the longest survival times in pig-to-nonhuman primate (NHP) xenotransplantation models using transgenic pigs as donors, as well as the possibility of growing humanized organs in pigs using blastocyst complementation [6, 7].

A role for NK cells in the rejection of cross-species and allogeneic hematopoietic stem cell transplantation (hybrid resistance) was already reported in the 1980s [8, 9]. In contrast, the initiation and regulation of adaptive immune responses after solid organ transplantation by NK cells, promoting either rejection or tolerance, has been recognized only more recently [10–12]. As to xenotransplantation, the demonstration by Inverardi et al. of early xenogeneic cell-mediated events taking place at the interface between the endothelium of a discordant vascularized organ and the recipient's blood cells using *in vitro* experiments and ex vivo perfusion models has generated a particular interest in the role of NK cells [13, 14]. Following this inspiring and pioneering work performed during the early 1990s, several laboratories have studied the interactions of human NK cells and porcine endothelial cells (pECs) that result in endothelial cell activation and damage *in vitro*. In addition, an array of possible strategies to reduce the observed endothelial damage caused by NK cells during rejection of vascularized xenografts has been put forward culminating so far in the generation of HLA-E transgenic pigs [15], which have been used in different xenotransplantation models [16–20].

During the past 20 years of research on NK cell biology, the view of these cells has evolved from simple killers to a heterogeneous, complexly regulated cell population able to control viral infections, to perform tumor surveillance, and to modulate adaptive immune responses [21–23]. Phenotypically human NK cells are characterized by the expression of the neuronal-cell adhesion molecule N-CAM (CD56) and the lack of CD3. Moreover, based on their level of CD56 expression, NK cells are divided into two major subpopulations: CD56^dim^ NK cells, which are more cytotoxic and express high levels of the low affinity Fc-gamma receptor III A (Fc*γ*RIIIA, CD16); and CD56^bright^ NK cells, which are characterized by the secretion of high levels of cytokines and low expression or absence of CD16 [24–26]. Overall, NK cell function is tightly regulated by the balance between activating and inhibitory signals mediated by a variety of NK cell receptors and their respective ligands on potential target cells. Upon recognition of “altered or abnormal cells” by one or a combination of the following mechanisms, these target cells will undergo lysis [27]:

- Recognition through CD16 (Fc*γ*RIIIA) of Abs bound to the surface of target cells leading to their elimination by a mechanism referred to as antibody-dependent cell-mediated cytotoxicity (ADCC).
- Recognition of the lack of self-major histocompatibility complex (MHC) class I molecules on target cells by inhibitory NK cell receptors leading to direct NK cytotoxicity.
- Presence of upregulated activating ligands on the surface of target cells (e.g., MICA/B for the activating NK receptor NKG2D) leading to direct NK cytotoxicity.
- Interactions of FasL and TRAIL expressed on NK cells with Fas and TRAIL receptors expressed on target cells resulting in apoptosis of target cells.

All these potential mechanisms of activation, recognition, and elimination of target cells by NK cells, alone or in combination, induce the release of the content of their lytic granules (perforin, granzyme, and cytolysin). In addition, the production and secretion of proinflammatory cytokines, such as tumor necrosis factor (TNF) and interferon gamma (IFN*γ*), has a major impact on the shaping of adaptive immune responses. The ultimate goal of NK cell function is thus not only the specific destruction and removal of identified target cells but also the activation and/or regulation of other components of the cellular immune system [23, 25].

Several years have passed by since the last extensive reviews on the role of human NK cells in pig-to-human xenotransplantation [28–34]. We will therefore summarize our knowledge and update the recent advances accomplished, including the mechanisms behind recruitment of NK cells into xenogeneic organs and vascularized composite tissues perfused with human blood, the testing of therapeutic strategies designed to provide protection against recognition and destruction of xenografts by human NK cells, and how regulatory T cells and tolerogenic dendritic cells (DC) may modulate NK cell xenoreactivity.

## 2. Recruitment of Human NK Cells to Porcine Tissues

The endothelium forms the interface between the recipient blood circulation and the donor parenchyma and is therefore the first xenograft component encountered by the recipient immune system upon revascularization. NK cells infiltrate xenogeneic tissues as demonstrated in rat heart or pig kidney ex vivo perfusion models using human blood [13, 35], in guinea pig- and hamster-to-rat small animal xenotransplantation models [36, 37] and pig heart-to-baboon and pig kidney-to-cynomolgus large animal transplantation models [38–41]. However, more recent pig-to-NHP models using transgenic and/or KO pigs and different immunosuppressive protocols identified only small numbers of infiltrating NK cells [42]. In the pig-to-human combination, the perfusion of pig lungs and limbs with human blood lead to the sequestration of human NK cells [18, 20]. Less NK cell infiltration was noted in pig hearts during whole blood perfusions [19].

Human NK cells express several chemokine receptors which are active under physiological and under inflammatory conditions [43]. However, only little is currently known on the role of chemokines during recruitment of NK cells to xenografts. A mouse heart and islet allotransplantation model supported the notion that chemokines act via CXCR3 in the recruitment of lymphocyte subsets including NK cells [44]. On the other hand, Chen and collaborators showed the relevance of MCP-1 (also known as CCL2) in the recruitment of NK cells into concordant mouse-to-rat xenografts [45]. Nevertheless, rodent xenotransplantation models do not sufficiently mirror the species compatibilities of ligand-receptor interactions between human and pig. Contact of human whole blood with pECs led to the secretion of human IL-6, CCL3, CCL4, CCL5, CCL11, and CXCL8. Moreover, binding of human natural non-*α*Gal xenoreactive Abs (XenoAbs) led to pEC activation and concomitant release of porcine chemokines and proinflammatory modulation of their surface receptors [46–49]. Chemokines such as CXCL8 secreted from activated pEC act on human polymorphonuclear neutrophils, preferentially through CXCR2 and PAF receptors, resulting in further secretion of chemokines and cytokines that can activate human NK cells [50].

Freshly isolated or IL2-activated, polyclonal NK cells were incubated with resting or activated, primary or immortalized pECs to further study NK cell responses against pECs. Various protocols were applied to analyze the different steps of recruitment *in vitro*, including under static and dynamic conditions simulating physiological shear stress [30, 51]. In our laboratory, primary and SV40-immortalized pECs derived from bone marrow (2A2) or aorta (PAEC, PEDSV.15) were used. All cell lines constitutively express von Willebrand factor, LDL receptor, and swine leukocyte antigen (SLA) class I, while SLA class II is expressed upon pig IFN*γ* but not upon human IFN*γ*-, pig or human TNF-stimulation [52]. In addition, pECs express the following adhesion molecules: PECAM-1 (CD31), E-selectin (CD62E), P-selectin (CD62P), and vascular cell adhesion molecule-1 (VCAM-1, CD106) [52, 53].

Although the methodological differences make it somewhat difficult to directly compare the results obtained by different research groups, the most dominant receptor-ligand interactions for the recruitment of human NK cells to pECs have been elucidated (Table 1). Initial *in vitro* assays performed under static conditions demonstrated the ability of NK cells to adhere to both resting pECs as well as TNF-activated pECs [54–58]. These studies using peripheral blood mononuclear cells (PBMC) also demonstrated a role for interactions between human VLA-4 (CD49d/CD29) and porcine VCAM-1 (pVCAM-1), the importance of which was subsequently confirmed using purified human NK cells [59, 60]. An even more pronounced role of these molecules was later shown in assays under physiological shear stress [53] with specific blocking of either the human *α*4 integrin (CD49d) or pVCAM-1 resulting in 75% reduction of adhesion of freshly isolated or activated NK cells to pEC. A significant role was also demonstrated for the *β*2 integrin LFA-1, which is expressed on human NK cells, by using blocking antibodies against both subunits, CD11a and CD18, respectively [53, 60, 61]. In addition, human L-selectin (CD62L), which mediates rolling, was required for human NK cell adhesion to pECs under physiological shear stress [53]. Concurrent *β*2 integrin, VLA-4, VCAM-1, and L-selectin blockade completely inhibited lymphocyte attachment [62].

As to the transendothelial migration (TEM), an initial study by Hauzenberger et al. reported a strong reduction of human NK cell TEM across pEC monolayers when blocking pVCAM-1 [63]. Consequently, we could show a role for pVCAM-1 in the actual TEM by using a model that separates adhesion from TEM [64]. With the same model, it was also demonstrated that *β*2 integrin (CD18) blocking inhibits both adhesion and TEM. However, the most important receptor on human NK cells specifically mediating TEM across pECs seems to be CD99 [64]. Furthermore, whereas homotypic CD31 interactions are very important for TEM of human leukocytes across human endothelial cells, blocking of CD31 did not influence adhesion or TEM in the pig-to-human combination [32, 64]. This finding agreed with the reported incompatibility between human and porcine CD31 [65]. Finally, one group reported that oxidative stress affects NK cytotoxicity and adhesion to pECs, mainly by reducing the expression of integrins, CD11b, and CD29, on NK cells, and the expression of E-selectin on pECs [66, 67]. Yet, ischemia-reperfusion injury and oxidative stress can be minimised in elective xenotransplantation in contrast to allotransplantation using deceased donor organs which often necessitates cold ischemia during transport of the organ.

In summary, NK cells are recruited to xenografts, perfused organs, or endothelial cell (mono) layers as shown in different models. *In vivo*, NHP NK cells can infiltrate pig organs to a certain degree, whereas ex vivo perfusion and *in vitro* experiments confirmed compatibilities of human and pig adhesion molecules allowing human NK cell recruitment. Molecular incompatibilities on the other hand lead to the activation of both pig endothelium and human NK cells, with consequent proinflammatory chemokine and cytokine production by both cell types. Further *in vivo* investigations using blocking antibodies to key adhesion molecules involved in the recruitment of human and NHP NK cells to pig endothelium, specifically targeting molecules like porcine CD106 (VCAM-1) and human/NHP VLA4 are warranted. In contrast, knocking out pig VCAM-1 to produce transgenic pigs might not work since this approach proved to be lethal in the mouse [68].

## 3. Recognition and Destruction of Pig Endothelium by Human NK Cells

**Figure 1:**
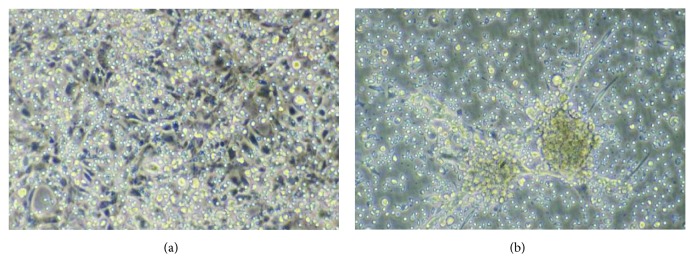
NK cytotoxicity against 2A2 pig endothelial cells. (a) Monolayers of porcine endothelial cells (pEC) were cultured to confluence and a suspension of IL2-activated purified polyclonal NK cells (bright round cells) was added on top of the monolayer, always in the absence of human sera. (b) After 4 hours of coculture with IL2-activated NK cells, the pEC monolayer was destroyed. Pictures were taken with a 200x magnification.

Adhesion of human NK cells to pECs *in vitro* leads to endothelial cell activation and eventually to endothelial cell damage (Figure 1). Malyguine et al. first reported morphological changes on pEC monolayers, the appearance of gaps, and the induction of a procoagulant state by human NK cells [69, 70]. Human NK cells activate pECs in a cell contact-dependent manner, characterized by the induction of E-selectin and IL8 via an NF-*κ*B-dependent pathway; the addition of IgG-containing XenoAbs further enhanced pEC activation and NK cell cytokine secretion (IFN*γ* and TNF) [71, 72]. Several groups, including our study [73], observed a role of human NK cells in both non-MHC restricted direct cytotoxicity and ADCC against pECs *in vitro*. The majority of these reports was published in the mid-1990s and has been reviewed in detail before [14, 29, 32]. Xenogeneic NK cytotoxicity against pECs can be increased by activation with human IL2, IL12, or IL15, whereas IL8 and IL18 have no effect [74]. The precise role of oligosaccharides, including the *α*Gal epitope, in the direct recognition of pECs by NK cells remains controversial (see Sections 3.1 and 4.1). In addition, human neutrophils recognize pEC independently of natural Abs and C' leading to endothelial activation, associated with increased cell surface expression of VCAM-1 and P-selectin and enhanced NK cytotoxicity [75]; the same might be true for monocytes although it has not been addressed directly. Although the main focus of the present review is on interactions between human NK cells and porcine endothelium, it has also to be mentioned that human NK cells are able to lyse porcine chondrocytes, islets, and embryonic brain-derived cells, via similar mechanisms as described for the destruction of endothelial cells [76–78].

### 3.1. ADCC against Pig Endothelium Mediated by Xenoreactive Antibodies

Natural or induced XenoAbs deposited on the graft endothelium can be recognized by Fc-receptors (FcRs) on effector cells, including NK cells, causing ADCC. Importantly, deposition of natural XenoAbs of IgG_1_ and IgG_2_ subclasses occurs on both wild-type (wt) and *α*1,3-galactosyltransferase knockout (GalT-KO) pECs, whereas IgG_3_ deposition was only detected on wt pECs [47]. NK cells express predominantly Fc*γ*RIIIa (CD16) recognizing IgG_1_ and IgG_3_, and, less efficiently, IgG_2_ [79]. ADCC involves the release of the contents of cytotoxic granules and the expression of death-inducing cell surface molecules (FasL, TRAIL) by NK cells. In general, xenogeneic ADCC depends, in addition to the class and subclass of IgG, on the density and stability of the Ag expressed on the surface of the target cell; XenoAbs' affinity for the Ag and FcR-Ab binding affinity. Other innate immune cells including monocytes, macrophages, neutrophils, eosinophils, and dendritic cells can eliminate Ab-coated target cells through phagocytosis and, to a lesser degree, ADCC via their FcR [80].

Human anti-pig ADCC was originally described by Watier et al. in pECs exposed to human peripheral blood mononuclear cells (PBMC) in the presence of human serum, while it could be prevented by the removal of IgG by immune absorption [81]. Moreover, blocking with anti-CD16 Abs abolished ADCC without affecting direct cytotoxicity [81]. Interestingly, no significant cytotoxicity was found in ADCC assays using normal sera or sera from diabetic patients, PBMC as effector cells and porcine islet cells as targets [82]. Moreover, Kumagai-Braesch et al. showed that ADCC was stronger in the presence of purified *α*Gal-specific Abs or anti-pig Abs present in the serum of xenoimmunized patients [76]. Enzymatic removal of *α*Gal from pECs reduced the binding of IgG, most pronounced for IgG_2_. However, it did not provide resistance against human IgG-dependent cytotoxicity indicating that *α*Gal was not the only xenogeneic epitope responsible for xenogeneic ADCC [83]. These findings were later confirmed by us using GalT-KO pECs [47].

**Figure 2:**
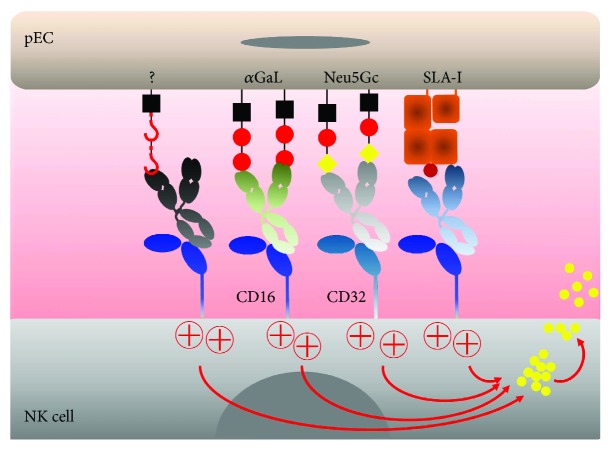
NK cell-mediated destruction of pig endothelial cells by recognition of human anti-pig antibodies (ADCC). Preformed natural XenoAbs circulating in the blood, mainly directed against *α*Gal but also other sugar antigens such as Neu5Gc, bind to pig endothelial cells with their Fab portion. The Fc-fractions of the antibodies are recognized by the FcRs located on the surface of NK cells, for instance, CD16 (Fc*γ*RIIIa) triggering the signaling cascade that leads to NK cell degranulation. The release of their lytic granules containing granzymes and perforin leads to target cell destruction, in this particular context, pig endothelial cells lysis, a process known as antibody-dependent cell-mediated cytotoxicity (ADCC). Alternatively, induced anti-SLA class I antibodies (far right) are recognized by NK cells via CD16, also leading to ADCC. *α*Gal: alpha Gal xenoantigen; HD Ag: Hanganutziu-Deicher antigen; Neu5Gc, SLA-I: swine leukocyte antigen class I.

The generation of human C' regulatory protein transgenic and GalT-KO pigs has largely overcome hyperacute rejection (HAR) in NHP models [3, 84, 85], illustrating the predominant role of *α*Gal. Nevertheless, acute XenoAb-mediated rejection directed against non-*α*Gal Ags still occurred in a pig-to-baboon heart transplantation model [86]. In human serum, some of these non-*α*Gal XenoAbs that induce E-selectin expression on pECs and complement C5b-9 deposition [48] recognize the Hanganutziu-Deicher Ag. This Ag is characterized by a terminal *N*-glycolylneuraminic acid (Neu5Gc) generated by conversion of the activated sugar donor CMP-Neu5Ac into CMP-Neu5Gc by cytidine monophosphate-*N*-acetylneuraminic acid hydroxylase (CMAH) [87–90]. Furthermore, an uncharacterized saccharide on pEC and synthesized by the porcine enzyme beta1,4 *N*-acetylgalactosaminyltransferase (B4GALNT2) is recognized by human XenoAbs [33]. The expression of Neu5Gc and lack of the corresponding natural XenoAbs in NHP, and the fact that deletion of CMAH in GalT-KO pigs increases NHP antibody binding, renders the interpretation of results obtained in pig-to-NHP models difficult [89, 91]. Recently, the non-*α*Gal problem was further addressed by the production of triple-KO pigs lacking the GalT, CMAH, and B4GALNT2 genes in order to abrogate the expression of *α*Gal, Neu5Gc, and the other unknown XenoAg, respectively [91]. Overall, pECs from these triple GalT•CMAHP•B4GALNT2-KO pigs did not support human natural XenoAbs-binding, but ADCC experiments have not been reported so far. Concerning XenoAbs directed against membrane proteins, some anti-HLA antibodies present in the serum of sensitized transplant patients cross-react with SLA class I, which has also been successfully knocked-out in pigs recently [92]. Furthermore, anti-porcine CD9, CD46, CD59, and EC protein C receptor XenoAbs were induced in a pig-to-NHP cardiac xenotransplantation model [93]. In conclusion, genetic modifications have substantially reduced or even eliminated the recognition of pECs by natural XenoAbs. However, the recognition of induced non-*α*Gal XenoAbs by NK cells remains to be addressed in ADCC experiments (Figure 2).

### 3.2. Receptor-Ligand Interactions Involved in Direct Xenogeneic NK Cytotoxicity against pECs

**Figure 3:**
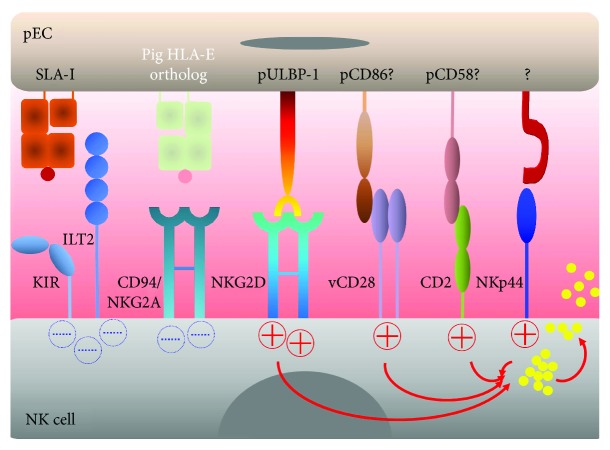
Receptors and ligands involved in pig endothelial cells lysis by human NK cells. There is a tight balance between activating and inhibitory signals that control NK cytotoxicity. The activating NK receptors NKG2D and NKp44 bind to their pig ligands: pULBP-1 and an unidentified molecule, respectively, and trigger lytic granule release (shown by red arrows and yellow circles). A role of CD2 and variant CD28 in facilitating NK cytotoxicity has been described in NK subpopulations, potentially by interacting with porcine CD58 and CD86, respectively. The inhibitory NK receptors, KIR, ILT2, and CD94/NKG2A, poorly recognize porcine MHC-I molecule (SLA-I) including the pig ortholog for HLA-E leading to a lack of inhibitory signals (in dotted blue) and NK cell activation.

Freshly isolated, as well as IL2-activated, human NK cells are able to recognize and destroy pECs of different anatomical origin, even in the absence of human XenoAbs indicating that the balance between activating and inhibitory receptors is disrupted. The major ligands recognized by inhibitory NK cell receptors are MHC class I molecules [94]. The human inhibitory killer cell immunoglobulin-like receptors (KIRs), KIR2DL2/2DL3, KIR2DL1, and KIR3DL1, are specific for the HLA-C1, HLA-C2, and HLA-Bw4 supratypes, respectively [24]. Another important inhibitory receptor on NK cells is immunoglobulin-like transcript 2 (ILT2) that also interacts with MHC class I, both classical and nonclassical [24, 95], and CD94-NKG2A recognizing HLA-E. Amino acid residues critical for the binding to human inhibitory NK cell receptors are altered in SLA class I as compared to HLA class I. Therefore, SLA class I cannot efficiently transmit inhibitory signals to human NK cells [96]. However, this incompatibility may be able to be at least partly overcome in situations where SLA-I expression is increased, such as following pEC activation by TNF or IL1 [59, 97]. Nonetheless, SLA class I molecules seem at least much less efficient compared to HLA class I in inhibiting human NK cells (Figure 3).

Among the known activating NK cell receptors [95], at least three are involved in NK cytotoxicity against pECs, CD2, NKp44, and NKG2D. Early studies showed a reduction of anti-pig NK cytotoxicity by specific blocking of CD2 on IL2-activated PBMC [58]. This effect was attributed to NK cells and not to T cells because blocking of human CD3 had no effect [58]. These initial results were recently confirmed by Kim et al. using purified NK cells and the same blocking strategy with anti-human CD2 Ab. However, the reduction in NK cytotoxicity and production of TNF and IFN*γ* by NK cells were not complete [98]. As to the potential pig ligands of CD2, that is, orthologs of CD58 (LFA-3) and CD59, blocking with anti-pig CD58 efficiently inhibited lysis of porcine targets by human PBMC to the same extent as anti-CD2 [98, 99]. Blocking of the adhesion molecule LFA-1 (CD11a/CD18) as well as of CD16, CD8, and CD57 on NK cells did not inhibit NK cytotoxicity against pECs [58] (Figure 3).

A role of NKp44 and NKG2D was demonstrated by reversal of NK cytotoxicity against pECs in the presence of blocking Ab, whereas other NK receptors including NKp30, NKp46, 2B4, CD49d, and CD48 were not involved [98, 100]. Of note, complete protection of pECs against cytotoxicity was only achieved when combinations of anti-NKp44 and -NKG2D or anti-CD2 and -NKG2D blocking Abs were used [98, 100]. As to the potential pig NKG2D ligands, we identified the ortholog of ULBP-1 (pULBP-1) and showed that it bound to human NKG2D, whereas pMIC2, another NKG2D ligand, was not involved in pEC destruction [101]. Interestingly, human serum-induced pULBP-1 on pECs, whereas treatment with either pig or human TNF or human cytomegalovirus infection of pECs led to a reduction of its expression [102]. Subsequently, Tran et al. detected an additional ligand of human NKG2D in porcine cells, the precise nature of which still remains unknown [103] (Figure 3).

The contribution of the costimulatory pathway CD28-CD80/CD86 to NK cytotoxicity against pECs has also been analyzed. A variant form of CD28 (vCD28) is expressed in subpopulations of NK cells. On the other hand, porcine cells including pECs and fibroblasts express pCD80/CD86, both constitutively and following exposure to T and NK cells [104, 105]. Blocking with a species-specific anti-pig CD86 antibody reduced xenogeneic NK cytotoxicity, whereas blocking pECs with anti-human CD80, CD86, and CD154 did not show any effect [105], indicating that interactions between vCD28-pCD86 are preserved across the species barrier [106]. In addition, blocking the vCD28-pCD86 pathway delayed xenograft rejection by inhibiting T and NK cell activation in a small animal cell transplantation model [107]. However, vCD28 has not been directly tested in the pig-to-human xenogeneic context (Figure 3).

### 3.3. Effector Mechanisms of Xenogeneic NK Responses

NK cytotoxicity is characterized by the pH-dependent release of perforin from lytic granules. Consequently, perforin assembly leads to pore formation in the target cell membrane and necrotic cell death. Moreover, these channels also enable other granule components including granzymes and granulysins to enter and to induce caspase activity and apoptotic cell death. Alternatively, apoptosis of target cells can be initiated by death receptor pathways including interactions between FasL expressed on NK cells and Fas on target cells. The latter mechanism does not play a role in xenogeneic NK cytotoxicity, because cross-species signaling between human FasL on NK cells and porcine Fas was only demonstrated using transfected porcine PK15 targets cells overexpressing pig Fas [108]. In contrast, a role of perforin and granzymes in human anti-pig NK cytotoxicity was demonstrated *in vitro* by the group of Nakajima and our own group using compounds that disturb the acidification of lytic granules (concanamycin A/B and ammonium chloride) and Ca^2+^ chelators that inhibit perforin polymerization [109–111]. A pan-caspase inhibitor prevented the lysis of pECs by human NK cells only partially in the absence of human serum [110], whereas specific caspase inhibitors demonstrated that only caspase-3 and -8, but caspase-1, are involved in ADCC mediated by XenoAbs [112]. Taken together, perforin/granzyme-dependent apoptosis and osmolysis, but not the FasL death receptor pathways, are implicated in human NK cytotoxicity against pEC. Finally, NK cells, beyond their function as cytotoxic effector cells, may initiate and regulate adaptive immune responses, thereby promoting either rejection or tolerance, as shown in solid organ allotransplantation [10–12]. Very little is so far known in the field of xenotransplantation. One study reported that NK cells eliminate cellular xenografts in a pig-to-mouse model via IFN*γ* but independently of perforin [113]. Another study showed that marginal zone B cells need help from NK cells to produce XenoAbs, a process that is independent of T cells and neither requires cytotoxicity nor IFN*γ* production [114].

In conclusion, the large majority of our knowledge on the mechanisms leading to human NK cell-mediated porcine endothelial cell recognition and destruction was generated *in vitro*. The role of NK cytotoxicity in pig-to-NHP xenograft rejection remains to be addressed more closely. It is likely, although not yet experimentally proven, that the immunosuppressive protocols currently used also inhibit NK cell functions (see below). However, as shown in some *in vivo* models, NK cells are also involved in promoting acquired xenoresponses or destruction of cellular xenograft mediated by cytokine production. It would be therefore of interest to study the effect of long-term NK cell depletion in preclinical *in vivo* models.

## 4. Strategies to Protect the Porcine Endothelium from NK Cytotoxicity

As expected, once that the major molecules involved in the interactions between human cells and pECs were characterized, the next step was to investigate whether it is feasible to manipulate these interactions or to identify inhibitory mechanisms to reduce the activity of human NK cells against pECs. A broad summary of successful strategies shown to reduce these interactions is summarized in Table 2. Despite the identification of the major adhesion molecule interactions responsible for NK cell binding to pECs and the availability of commercialized monoclonal Abs blocking, for example, VLA4 (natalizumab), there are essentially no studies trying to test adhesion blocking approaches *in vivo* or ex vivo [14, 115]. Thus, we will pay special attention to approaches to control human anti-pig NK cytotoxicity: (i) the potential of carbohydrate modifications such as *α*Gal knockout to prevent NK cell responses; (ii) manipulations of apoptotic pathways and dextran sulfate; and (iii) expression of human MHC class I molecules on pECs binding to inhibitory human NK cell receptors that initiate immunoreceptor tyrosine-based inhibitory motif- (ITIM0-) dependent negative signaling pathways [116].

### 4.1. Removal of *α*Gal Epitopes and Modification of Other Sugar Antigens

The removal of *α*Gal from pigs has been a great advance in the field of xenotransplantation as HAR was avoided in pig-to-baboon xenotransplantation models [117, 118]. The contribution of *α*Gal to NK cell-mediated responses in the absence of natural or induced anti-*α*Gal Abs remains controversial. Some groups reported that human NK cells directly recognize oligosaccharide ligands expressed by xenogeneic cells [87, 106, 119, 120]; carbohydrate remodeling of pECs, for instance, increased the susceptibility to human NK-mediated lysis as demonstrated by transfection of *α*(1,2)-fucosyltransferase in pECs [119]. Christiansen et al. reported interactions between the human NK receptor NKRP1A and *α*Gal [121]. In contrast, we and others could not confirm the role of *α*Gal as a dominant cytotoxicity-inducing NK target molecule when testing NK cytotoxicity against GalT-KO or GalT RNA-silenced pEC [46, 122–126]. A significant difference between these latter and earlier experiments was that there was no need to treat the target cells with either galactosidase or blocking reagents [46, 122–126]. NK cell-mediated ADCC was reduced by 30 to 70% in the absence of *α*Gal expression on pECs, whereas direct xenogeneic lysis mediated either by freshly isolated or IL2-activated human NK cells or the NK cell line NK92 was not reduced [46, 124]. Nonetheless, full elimination of *α*Gal from pECs prevented complement-induced lysis (up to 86%) and ADCC (from 30–70%) but not direct xenogeneic human NK cytotoxicity mediated by freshly isolated, IL2-activated NK cells or the NK-92 cell line [46]. Conversely, NK cell-pEC adhesive interactions were not reduced [46]. In addition, *α*Gal-independent interactions between human NK cells and pECs triggered an intracellular Ca^2+^ rise in pECs, followed by an upregulation of P-selectin and VCAM-1, and NK cell activation resulting in increased expression of perforin and cytotoxicity [122]. Transgenic expression of *α*Gal on primary human aortic endothelial cells, as shown by He's group did neither trigger NK cytotoxicity nor adhesion [123]. However, these results do not completely rule out a role for *α*Gal in NK recognition; it may be necessary but not sufficient to interfere with *α*Gal to overcome xenogeneic NK responses. The recent generation of KO pigs for multiple saccharide XenoAgs resulted in no remaining binding of human Abs [91], but the direct effect on NK cell responses has not been tested yet.

### 4.2. Manipulation of Apoptotic Pathways

Two approaches to protect pig endothelial cells from NK cytotoxicity by genetic engineering of the apoptotic pathways have been explored: (i) overexpression of FasL in order to induce lysis of activated Fas-expressing human effector cells including NK cells; and (ii) overexpression of antiapoptotic proteins such as Bcl-2 and A20 to counter-balance proapoptotic signals induced by NK cells.

Transfection of pig epithelial cells (PK15) with human FasL provided partial protection against human NK cytotoxicity [108, 127]. In addition, overexpression of porcine FasL reduced the susceptibility to lysis by IL2-activated human NK and T cells by inducing apoptosis [128]. In our hands, expression of human FasL on pECs did not provide protection against human NK cytotoxicity, although apoptosis of human NK cells was observed. Moreover, human FasL expression had no effect on NK cell adhesion to pECs. In contrast, NK cell migration through pECs and chemotaxis of human polymorphonuclear cells were strongly increased by the expression and cleavage of soluble FasL [129], consistent with earlier reports [130].

Late in the ‘90s, considerable levels of the antiapoptotic proteins Bcl-2 and A20 were found in the graft endothelial cells in rodent xenografts that were “accommodated” [131]. Overexpression of Bcl-2 in pEC did not provide protection against human NK cytotoxicity in our hands [110]. In contrast, in another preliminary study by Nakajima et al. Bcl-2 expression in PK15 cells provided partial protection against apoptosis caused by human perforin/granzyme in ADCC assays or Fas/FasL interactions [112, 132]. Finally, transgenic pigs expressing A20 on their endothelial cells have been generated, but the protective role of A20 against human NK cytotoxicity was formally not yet studied [133, 134].

### 4.3. Dextran Sulfate Protects Pig Endothelial Cells from NK Cell-Mediated Cytotoxicity

Another approach to protect pECs from NK cytotoxicity explored the use of low molecular weight dextran sulfate, an analog of proteoglycans that are shed from pECs upon activation. Indeed, there was a protective effect when pECs, activated or not with pig TNF, were exposed to human NK cells in the presence of dextran sulfate. This protection was specific for pECs because when the prototypical NK target cell K562 was preincubated with dextran sulfate, the NK cytotoxicity was not affected [135].

### 4.4. Expression of Classical HLA Class I Molecules HLA-Cw4 and HLA-Cw3 in Porcine Endothelial Cells Inhibits NK Cytotoxicity

Certain HLA class I supratypes bind to inhibitory killer cell immunoglobulin-like receptors (KIRs), for example, CD158a (KIR2DL1) and CD158b (KIR2DL2/3). Specific natural ligands for CD158a and CD158b are HLA-Cw4 and HLA-Cw3, respectively [24]. Transfection of pECs with plasmids encoding HLA-Cw4 and HLA-Cw3 led to partial protection from bulk human NK cytotoxicity and complete protection from NK clones with high expression of CD158a [136]. Taking this approach one step further, Sharland et al. reported that a porcine B-lymphoblastoid cell line transfected with HLA-Cw^∗^0304 gene constructs encoding genetically modified HLA-Cw3 unable to interact with CD8, inhibited both direct cytotoxicity and ADCC mediated by human NK clones expressing the appropriate CD158b inhibitory receptor while avoiding recognition by human CD8^+^ T cells [137, 138]. However, expression of both HLA-Cw3 and -Cw4 did not confer further protection. Intriguingly, the expression of HLA-Cw4 also reduced the adhesion of human NK cells to pECs [136, 139]. Finally, expression of HLA-B27 on pECs provided only moderate to low protection from NK lysis even when NK cells derived from HLA-B27 positive donors were tested. HLA-A2 expression did not protect from xenogeneic NK cytotoxicity [136].

### 4.5. Expression of HLA-G in Porcine Endothelium Inhibits NK Cytotoxicity and Adhesion

HLA-G is a nonclassical human MHC class I molecule with limited polymorphism compared to classical HLA class I alleles. In addition, HLA-G inhibits human NK cytotoxicity without inducing T cell alloresponses. The human ligands for HLA-G are KIR2DL4 (CD158d) and immunoglobulin-like transcripts 2 and 4 (ILT2, ILT4). These are expressed at different levels on NK cells and other cells and therefore made this molecule attractive to study in the context of pig-to-human xenotransplantation [140]. Initial work showed that transfection of pECs with HLA-G only had a modest protective effect on NK cytotoxicity and that the expression of ILT-2/LIR-1 on NK cells did not correlate with the HLA-G mediated inhibition [141–143]. Surprisingly, HLA-G reduced rolling adhesion of activated human NK cells on pECs adding a new function to this nonclassical HLA class I molecule [144]. Finally, the group of Chen et al. showed that pECs were protected from human NK cytotoxicity by soluble HLA-G_1_ [145]. In contrast to HLA-E (Section 4.6.), HLA-G protects porcine cells from lysis by human NK cells through a CD94/NKG2-independent pathway [142, 146, 147].

### 4.6. Expression of HLA-E in Porcine Endothelium Inhibits Xenogeneic NK Cytotoxicity

HLA-E is another nonclassical HLA class I molecule restricted to only two functional variants making it attractive to use in xenotransplantation. HLA-E is recognized by the inhibitory NK cell receptor CD92/NKG2A and by the activating receptor CD92/NKG2C, although with 5–10 fold lower affinity [140, 148]. In fact, HLA-E provided partial protection of transfected pECs from polyclonal xenoreactive human NK cell populations and total protection when NK clones expressing high levels of NKG2A were used as effectors [149, 150]. In contrast to what was observed for HLAC-Cw4 and HLA-G, HLA-E had no effect on the adhesion of human NK cells to pECs [151], a result which was recently confirmed in an elegant *in vitro* flow system under dynamic conditions using pEC stemming from HLA-E transgenic pigs [20].

These *in vitro* results, in combination with the fact that HLA-E is relatively nonpolymorphic and thus of low alloreactivity, stimulated the generation of double HLA-E/human *β*2microglobulin transgenic pigs in a collaborative project with the Munich group [15]. Indeed, pECs derived from these transgenic animals showed partial protection against human NK cytotoxicity, depending on the level of expression of CD94/NKG2A on NK cells, and lower production of IFN*γ* by NK cells in response to pECs [15].

In order to take these studies a step further, several ex vivo xenoperfusion models have been established allowing investigations on the interactions between human blood and pig tissues directly. Collaborating with a multidisciplinary team in Berne, we explored a pig limb perfusion system using human blood [16]. Compared to wild-type pig limbs, humoral xenoresponses were reduced in double transgenic pigs expressing human CD46, a C' regulatory protein, and HLA-E. Moreover, NK cells were quickly removed from the circulating blood infiltrating the muscle tissue. Slightly delayed NK cell recruitment and reduced tissue infiltration were observed in perfused HLA-E/hCD46 double transgenic pig limbs [18]. The expression of HLA-E in transgenic pigs has also been tested in ex vivo lung and heart perfusion with human blood, and showing similar results in terms of reduced tissue damage, most likely linked to a reduction of NK responses [19, 20]. Since the expression of the HLA-E receptor CD94/NKG2A on NHP NK cells and functional inhibition of NHP NK cells by HLA-E remains to be addressed experimentally (Section 6), it is hard to judge whether currently used pig-to-NHP models are appropriate to test the role of HLA-E in inhibiting human anti-pig xenogeneic NK cell responses *in vivo*.

### 4.7. Effect of Immunosuppressive Drugs and Biologicals on NK Cells

The mechanisms of T and B cell inhibition mediated by conventional immunosuppressive drugs (ISD) and biologicals used in transplantation medicine are well known. As to NK cells, the literature reports a variety of specific effects of ISD on NK cells with to some extent conflicting conclusions [152–160]. A comprehensive comparative study confirmed that corticosteroids are potent inhibitors of NK functions including ADCC, direct NK cytotoxicity, and IFN*γ* production (own unpublished data). In addition, NK cytotoxicity was inhibited by the highest therapeutic doses of cell cycle (mycophenolate mofetil) and mTOR inhibitors (everolimus). As to calcineurin inhibitors, cyclosporine inhibited direct cytotoxicity, whereas tacrolimus reduced both, ADCC and direct cytotoxicity. Little is known so far on the effect of biologicals or monoclonal antibodies on NK cell function, for example, tocilizumab (anti-IL6R), infliximab (anti-TNF), and natalizumab (anti-VLA4) [161]. Moreover, monoclonal antibodies that block costimulation such as anti-CD154 and anti-CD40 are being used in xenotransplantation models with great success [162], but their effect on NK cells is largely unknown. Expression of CD154 on NK cells upon IL2 stimulation increased NK cytotoxicity in one study [163], and human NK cells were shown to activate autologous human B cells via CD40-CD154 interactions [164]. On the other hand, treatment with an agonistic anti-CD40 reduced NK cell numbers in the circulation in one cancer study [165], but nothing has been published in the transplantation field. Taken together, it is very likely that the immunosuppressive protocols currently used in preclinical pig-to-NHP xenotransplantation models have an important inhibiting impact on NHP NK cells but this remains to be addressed in more detail in future studies.

As outlined in this chapter, several different strategies have been developed to control NK cell xenoresponses. In addition to the removal of *α*Gal and other oligosaccharide ligands of preformed anti-pig XenoAbs leading to the reduction of ADCC, the generation of HLA-E transgenic pigs is the most promising approach, although there is still not enough *in vivo* evidence to fully support the relevance of this strategy to protect pig xenografts from human NK cell-mediated injury. Finally, ISD, in particular corticosteroids, inhibit NK cells, but the effect of ISD, especially the new generation of biologicals on NHP/human NK cells, warrants further exploration. However, the “Holy Grail” would be to find ways to induce NK cell tolerance towards xenografts and to avoid ISD and their side effects.

## 5. Modulation of Xenogeneic NK Responses by Other Immune Cells

Emerging strategies to control “unwanted” NK cell-mediated immune responses in xenotransplantation not only include inhibition of NK cells or manipulation of their presumed targets (specifically pEC) by genetic engineering of cell surface molecule expression patterns but also the use of regulatory immune cells of recipient origin to induce transplantation tolerance.

One important approach to induce transplantation tolerance is the mixed hematopoietic chimerism model. The group of Sykes discovered that NK cells become specifically tolerant to donor cells in murine mixed allogeneic chimeras, whereas NK cell tolerance was associated with global unresponsiveness of the murine NK cells in the xenogeneic rat-to-mouse mixed chimerism model [166]. More recently, the same group studied the effect of mixed porcine chimerism on human NK cell phenotype and function, xenogeneic cytotoxicity, and IFN*γ* production, in a humanized mouse model with induced NK cell reconstitution. Interestingly, variable and partial human xenogeneic NK cell tolerance to pig cells was demonstrated in cytotoxicity assays [167]. The effect of this promising tolerance approach remains to be further explored and tested on NK cells in NHP models.

Whereas the various cell populations involved in transplant rejection and strategies on how to suppress them are well known, the cell populations involved in the induction and maintenance of transplant tolerance remain less well characterized. In particular, the respective and precise contribution of DC and T cells in cell-mediated xenograft rejection and tolerance induction is not yet fully dissected. Human DC effectively adhered to pEC and were activated by xenoantigens, resulting in highly efficient antigen presentation and proliferation of CD4^+^ T cells [168]. On the other hand, porcine + derived from huTRAIL transgenic pigs decreased human T cell proliferation significantly without any signs of apoptosis [169]. *In vitro* human CD4^+^CD25^+^ regulatory T cells suppressed indirect xenogeneic immune responses mediated by DC pulsed with porcine epithelial cells [170]. Moreover, human myeloid-derived suppressor cells were shown to inhibit anti-pig xenogeneic responses mediated by human NK cells and cytotoxic T lymphocytes (CTL) [171]. Finally, Ierino's group demonstrated that the use of pig DC with tolerogenic properties significantly reduced human T cell responses when used as stimulators in human lymphocyte proliferation assays *in vitro* [172].

Recently, we reported the efficiency of two different human monocyte-derived DC generated under tolerogenic conditions, that is, in the presence of IL10 and rapamycin, to control xenogeneic NK cells and CTL. Indeed, IL10-DC were able, at least *in vitro*, to decrease NK degranulation and intracellular IFN*γ* production in response to pEC. In addition, tolerogenic IL10-DC reduced xenogeneic CTL cytotoxicity in a haplotype-specific manner [173]. *In vivo*, mouse IL10-DC were used in a concordant rat-to-mouse islet xenotransplantation model, showing an overall increase of xenograft survival, the mechanisms of which are currently under investigation [174]. Furthermore, human regulatory T cells (Treg) have been shown to inhibit NK cells by several different mechanisms [175] and also to suppress xenogeneic immune responses [176]. Recruitment of human Treg to pECs depends on particular chemokine receptors (CXCR3 and CCR4) and integrins (CD18 and CD49d). *In vitro*, human Treg partially suppressed xenogeneic human NK cell adhesion to pECs, as well as xenogeneic cytotoxicity and degranulation [177]. Taken together, these results will help to develop new protocols to specifically regulate NK cell-mediated xenograft responses by using Treg and tolerogenic DC.

In conclusion, modulation of cellular immune responses by mixed chimerism induction and cell therapy has been tested successfully in both allotransplantation and xenotransplantation models. The mixed chimerism approach has even been successfully used in clinical trials but is still limited by the inherent toxicity of hematopoietic stem cell transplantation in NHP and humans. Strategies using Treg and tolerogenic DC to prevent graft rejection are attractive new frontiers that are now being translated to the clinic and to the field of xenotransplantation research.

## 6. The Role of NK Cells in Preclinical Nonhuman Primate Models

Whereas the molecular interactions between human NK cells and pig cells can be rather easily studied *in vitro*, it is much more difficult to draw firm conclusions on their relevance *in vivo*. Though very helpful for hypothesis-driven basic research, rodent models are often of limited value for human conditions. The translation of results stemming from these experiments to human clinical application remains largely elusive due to fundamental differences of the respective immune systems. While humanized mouse models and restoring physiological microbial exposure in mouse husbandry may provide some improvement [178, 179], progress in xenotransplantation research relies essentially on NHP models. Nevertheless, differences in NK cell biology between humans and baboons or cynomolgus monkeys should be taken into consideration when analyzing immune responses in pig-to-NHP xenografts. Early immunohistological studies have suggested that NK cells are involved in pig-to-NHP xenograft rejection, but these analyses suffered from unreliable staining techniques [38, 40]. *In vitro*, a number of groups have analyzed the phenotype, natural cytotoxicity, and antiporcine function of NHP NK cells [38, 40, 180–182]. Destruction of pig endothelium mediated by baboon NK cells via ADCC and direct cytotoxicity and spontaneously enhanced following IL2-activation was demonstrated and depended on CD2 and CD49d as shown by antibody blocking studies [40]. Furthermore, peripheral blood lymphocytes (PBL) from nonsensitized baboons spontaneously lysed pEC, which was inhibited by both anti-CD2 and anti-CD94 blocking. Reduction of galactosyl residues by galactosidase digestion decreased almost completely pEC lysis by nonsensitized, but not primed, baboon PBL [181]. Finally, baboon NK cells can be identified and isolated on the basis of a CD3^−^NKp46^+^CD8^dim^CD16^+/−^ or CD3^−^CD8^dim/−^CD16^bright^ phenotype and expressed CD56 upon IL2 activation. They exert very low spontaneous cytotoxicity against both human (K562) and pig target cells, but it can be significantly stimulated by IL2 activation [182].

Little is known as to the expression of other receptors on baboon NK cells including NKp30, NKp44, NKG2A, NKG2D, and KIR/CD158, while expression of KIR, CD94/NKG2A, and NKp80 has been demonstrated on rhesus monkey NK cells [183–185]. However, validation of cross-species reactivity of monoclonal rodent-anti-human antibodies used to phenotype NHP NK cells remains a problem. In summary, a better molecular and functional characterization of NHP NK cells is warranted for studying their role in porcine xenograft rejection using preclinical pig-to-NHP models.

## 7. Conclusions

Big advances characterize the past 25 years of xenotransplantation research and in particular the elucidation of the role of NK cells. Although the evidence for a role of NK cells in preclinical pig-to-NHP xenotransplantation models is still weak, it became clear that NK cell anti-pig responses most likely do play a role in xenograft rejection, especially early in the process. They are also involved in shaping xenoresponses mediated by the adaptive immune system. The molecular cross-talk between human NK cells and porcine endothelium that governs recognition and activation of NK cells as well as induction of ADCC via XenoAbs and direct perforin/granzyme-dependent cytotoxicity has been elucidated. Many of these interactions can be explained by either molecular cross-species incompatibilities or intact receptor-ligand interactions between pig ECs and human NK cells. The efforts to understand and to overcome human anti-pig NK cell responses have led to the generation of HLA-E transgenic pigs, which provide at least partial protection, and the advent of new strategies aiming at tolerizing NK cells. In our view, future studies on the NK cell biology of NHP are needed to further elucidate the role of NK cells in pig-to-NHP xenotransplantation preclinical models. To perform these studies and to go beyond the histological characterization of xenograft infiltration, species-specific tools such as antibodies and NK functional assays need to be established. Additionally, it would be of interest to explore *in vivo* the role of new ISD like costimulation blockers in NHP xenotransplantation models to prevent NK cell-mediated xenograft rejection and to induce graft tolerance.

## Figures and Tables

**Table 1:** Integrins and selectins and their ligands involved in NK cell recruitment to pig endothelium [30, 186].

Protein family	Name	CD name	Heterodimer	^∗^Receptor location	Ligands	Ligand CD name	Ligand location	Cross-species interaction
Integrins	*α*4	CD49d	*α*4/*β*1	NK	VCAM-1 fibronectin	CD106 —	pECs ECM	Yes Yes
			*α*4/*β*7	NK	VCAM-1 MadCAM-1	CD106 —	pEC EC (pEC?)	Yes U
	*α*6	CD49f	*α*6/*β*1	NK	Laminin	—	ECM	No
	*α*L, LFA-1	CD11a	*α*L/*β*2	NK	ICAM-1 ICAM-2	CD54 CD102	pECs pECs	Yes U
	*α*M, Mac-1	CD11b	*α*M/*β*2	NK	ICAM-3 RAGE	CD50 —	pECs ST	Yes U
	*β*1	CD29	*α*4/*β*1	See above				
			*α*6/*β*1	See above				
	*β*2	CD18	*α*L/*β*2	See above				

^†^Integrin dimers	VLA-4	CD49d/CD29	*α*4/*β*1	See above				
	VLA-6	CD49f/CD29	*α*6/*β*1	See above				
	LPAM-1	—	*α*4/*β*7	See above				

Selectins	E-selectin	CD62E	—	pECs	PSGL-1 sialyl LewisX Sialophoryn	CD162 CD15s CD43	pECs NK NK	No U U
	L-selectin	CD62L	—	NK	Mucosialin MadCAM-1 GlyCAM-1	CD34 — —	pEC pECs	Yes
	P-selectin	CD62P	—	pECs	PSGL-1 Sialyl LewisX	CD162 CD15s	NK NK	No U
	pMIC2?	pCD99?	—	pECs	MIC2, E2	CD99	NK	Yes

Adhesion molecules	PECAM-1	CD31	—	pECs	PECAM-1	CD31	NK	No

^∗^Cell on which the molecule is expressed. ^†^Integrins exist as heterodimers and are composed of one *α* and one *β* unit. CD: cluster of differentiation; ECM: extracellular matrix; NK: human natural killer cells; pEC: pig endothelial cells; ST: several tissues; U: unknown.

**Table 2:** Proven strategies to overcome NK cytotoxicity against pig endothelial cells.

Target	Approach	NK source	Effect	Reduction (%)	Ref
*α*Gal GT	*In vitro* knockout pEC lines	IL2-NK cells, NK92 Fresh NK cells, Fresh NK cells	No effect CMC ↓ ADCC ↓ nAb/CML	NA 77–90 86	[46, 126]
	Gal knockout pigs	IL2-NK cells, Fresh NK cells Fresh NK cells	No effect in CMC ↓ ADCC ↓ nAb/CML	NA 70 80	[47, 124]
	siRNA in pEC	NK92 cell line	No effect CMC ↓ ADCC	NA 30–40	[125]

Masking of sugar xenoantigens	Transfections of *α*(1,2)-fucosyltransferase in pECs	Fresh NK cells	↓ CMC	47	[119]
	Treatment of pECs with DXS	NK92	↓ CMC inh. C' deposition	25–47	[135]

HLA class I molecules	HLA-E transfection in pEC	IL2-NK cells	↓ CMC	15–60	[150, 151]
	HLA-E transgenic pigs	IL2-NK cells	↓ CMC ↓ IFN*γ* production	8–30 40	[15]
	HLA-G1 transfection in pEC	IL2-NK cells, NK92 and NK cell clones	↓ CMC ↓ rolling/adhesion No effect in ADCC	20–45 25–75 NA	[141, 142, 150, 187]
	Soluble HLA-G1	NK92 Fresh PBMC	↓ CMC ↓ CMC	31–83 24	[145]
	HLA-Cw3 transfection in pEC	NK cell clones	↓ CMC	12–70	[136]
	HLA-Cw4 transfection in pEC	NK cell clones	↓ CMC	58	[139]
	HLA-B27 transfection in pEC	NK cell clones	↓ CMC	~30	[136]

Apoptosis induction	PK15, human FasL transfection	PBL	↓ ADCC	34–42	[108]
	PK15, human FasL transfection	PBL, LAK	↓ ADCC ↓ CMC	54 74	[127]
	pEC, human FasL transfection	IL2-NK cells	No effect CMC ↑ apoptosis NK cells	0	[129]
	pEC, pig FasL transfection	IL2-NK cells	↓ CMC ↓ FasL apoptosis	26 23	[128]

Apoptosis resistance	Bcl-2 transfection in PK15	PBL	↓ FasL apoptosis ↓ ADCC	62 50	[132]
	Bcl-2^mut^ transfections in pEC	IL2-NK cells	No effect CMC	0	[110]

ADCC: antibody-dependent cell-mediated cytotoxicity; *α*Gal GT: alpha1,3-galactosyltransferase; C': complement; CML: complement-mediated lysis, CMC: cell-mediated cytotoxicity; DXS: dextran sulfate; HLA: human leukocyte antigen; IFN*γ*: interferon gamma; inh.: inhibition; LAK: lymphokine-activated killer; NA: not applicable; nAb: natural antibody; PBL: peripheral blood lymphocytes; PBMC: peripheral blood mononuclear cells; pEC: porcine endothelial cells; PK15: pig kidney cell line; siRNA: short interfering RNA; ↓: reduction.

## Abbreviations

Ab: Antibody
ADCC: Antibody-dependent cell-mediated cytotoxicity
Ag: Antigen
*α*Gal: Alpha Gal xenoantigen
C': Complement
Fc*γ*R: Fc-gamma receptor
FasL: Fas ligand
GalT-KO: Knockout for the enzyme *α*1,3-galatosyltransferase
Grz: Granzyme
HAR: Hyperacute rejection
HLA: Human leukocyte antigen
IFN*γ*: Interferon gamma
KIR: Killer cell immunoglobulin-like receptor
KO: Knockout
MIC: MHC class I chain related protein
NK: Natural killer
NHP: Nonhuman primates
PBMC: Peripheral blood mononuclear cells
PBL: Peripheral blood lymphocytes
pECs: Porcine endothelial cells
SLA class I: Swine leukocyte antigen class I
TEM: Transendothelial migration
TNF: Tumor necrosis factor alpha
TRAIL: TNF-related apoptosis-inducing ligand
ULBP: UL-binding protein
VLA: Very late antigen
wt: Wild-type
XenoAbs: Xenoreactive antibodies.
